# Expanding unilateral cochlear implantation criteria for adults with bilateral acquired severe sensorineural hearing loss

**DOI:** 10.1007/s00405-019-05358-z

**Published:** 2019-02-27

**Authors:** Wendy J. Huinck, Emmanuel A. M. Mylanus, Ad F. M. Snik

**Affiliations:** 1 Department of Otorhinolaryngology, Head and Neck Surgery, Radboud University Medical Center, PO Box 9101, 6500 HB Nijmegen, The Netherlands; 2 Donders Department of Biophysics, Institute for Brain, Cognition and Behaviour, Radboud University, Nijmegen, The Netherlands

**Keywords:** Hearing loss, Cochlear implantation, CI indication criteria, Performance, Speech recognition, Quality of life

## Abstract

**Objectives:**

To report on a retrospective cohort study on the effects of expanding inclusion criteria for application of cochlear implants (CIs) on the performance 1-year post-implantation.

**Methods:**

Based on pre-implantation audiometric thresholds and aided speech recognition scores, the data of 164 CI recipients were divided into a group of patients that fulfilled conservative criteria (mean hearing loss at 0.5, 1 and 2 kHz > 85 dB HL and phoneme scores with hearing aids < 30%), and the remaining group of patients that felt outside this conservative criterion. Speech recognition scores (in quiet) and quality of life (using the NCIQ) of both groups, measured at 1-year post-implantation, were compared.

**Results:**

The group that felt outside the conservative criterion showed a higher phoneme score at 1-year post-implantation compared to the conservative group, suggesting that relaxed criteria have a positive influence on the speech recognition results with CI. With respect to quality of life, both groups significantly improved 1-year post-implantation. The conservative group showed a higher benefit on the advanced perception domain of the NCIQ. Based on their worse pre-implantation hearing, this was expected.

**Conclusions:**

The data suggest that relaxation of CI indication positively affects the speech recognition performance of patients with severe hearing loss. Both groups of patients showed a positive effect of CI on the quality of life. This benefit relates to communication skills and the subjective day-to-day functioning in society.

## Introduction

Cochlear implantation is a treatment for patients with deafness who do not profit from conventional hearing aids. A cochlear implant (CI) transforms the acoustic signal into an electrical signal that activates directly the auditory nerve fibers. In adults with acquired deafness, CIs provide open-set speech understanding in the majority of patients [1].

The audiological inclusion criteria for cochlear implantation differ over countries [2–5]. Since the first cochlear implantation, CI-technology and knowledge regarding surgery and fitting have continuously developed, leading to improved performance. In turn, this has lead to relaxation of the audiological implantation criteria. Whereas CI initially was meant to be a solution for patients with total deafness, it gradually evolved in a solution for patients with severe to profound hearing loss as well as for patients with a partial (high frequency) deafness [6, 7]. Luntz et al. [8] describes the initially stiff process of accepting cochlear implantation as a safe hearing solution in the early days. Today, unless the enormous amount of research showing the benefit of cochlear implantation, the indication procedure remains hard and inconvenient for many CI candidates [8]. Several studies have shown that the degree of functional residual hearing, pre-implantation, is correlated with CI performance [9–11]. The reason for this is that preoperative residual hearing is thought to act as a “trophic factor” that protects the spiral ganglion and/or the central auditory pathways from degeneration [10]. Without functional residual hearing, pre-implantation, for a prolonged period, the auditory neural system might be deprived as a result of a lack of auditory stimulation. This might even be the case by single-sided deafness. In a recent study [12], Cohen and Svirsky conducted a systematic review on the relationship between duration of unilateral deafness and speech perception outcomes after CI in adults with single-sided deafness. Although the effect found was rather small and additional research need to strengthen these findings, the authors point on the important implications suggesting that unilateral sound deprivation, even when the contralateral normal hearing ear still receives auditory input, may have a negative effect on the auditory processing.

The shift of (unilateral) inclusion criteria positively affects the overall performance with a CI [13], which might be owing to less auditory deprivation. Therefore, the need for relaxing the CI audiological indication criteria and, consequently, earlier implantation, is growing. It is suggested that in several countries, the indications for candidacy do—as a result of these shifting insights—no longer reflect the entire population of patients that should be considered for cochlear implantation.

So, what is the best CI indication and how strict should this be applied? There is a wide variability in CI indication criteria across the countries [1, 6, 14–18]. Vickers et al. collected information on indication criteria in 17 countries; in general, CI indication criteria were based on either speech recognition with conventional hearing aids (more functional) or based on the audiometric hearing loss or both. In The Netherlands [16] for example, patients are considered for cochlear implantation if the phoneme score, presented at normal conversation level and obtained with a well-fitted conventional hearing aid, is less than 50%, which equals a word score of 20% [16]. In the Netherlands, this 50% criterion is set in consensus by the Dutch CI centers. However, since Dutch CI centers are allowed to deviate from this criterion on individual basis [16], this criterion has gradually shifted towards 70% phoneme score (44% word score) in quiet.

This means that if there is insufficient benefit of acoustic hearing aids, a patient might become a candidate for CI, even when the hearing thresholds are not at a profound level. This is in contrast with some other countries that hold on to a more conservative approach. In Belgium, for example, the inclusion criterion is set at a phoneme scores of 30% (which equals a word score of 6%) or less and a hearing threshold (PTA, mean hearing loss at 0.5, 1 and 2 kHz) above 85 dB HL [17].

In several countries, audiometric hearing thresholds are the sole basis for CI inclusion; however, these thresholds do not always reflect the actual problems faced by an individual with severe hearing impairment [7, 19]. The factors of influence are, for example, the cause and duration of hearing loss, age at implantation, central auditory factors, cognition, motivation, position of the electrode, lifestyle, socio-economic factors, etc.[20–23] and this emphasizes the importance of an individual approach, taking such factors into consideration.

Another pitfall in CI indication is that criteria are often applied rigidly. Hearing loss might be progressive (e.g. genetic types) and, therefore, it is often not the question if the patient will receive an implant but rather when the patient will be implanted. Strict inclusion based on hearing thresholds might result in postponing cochlear implantation leading to a non-optimal result owing to auditory deprivation, while the patients’ level of social functioning remains limited until better hearing is achieved using CIs.

To study the effect of expanding the inclusion criteria, a retrospective cohort study is performed to analyze the effect of pre-implantation hearing level on CI performance.

Based on pre-implantation audiometric thresholds and speech recognition scores obtained with well-fitted conventional hearing aids, the data of a large group of CI recipients were divided into two groups: those patients that fulfilled conservative criteria (as applied in for example, Belgium) and the remaining group of patients that felt outside the conservative criterion but still inside the broadened Dutch inclusion criterion.

For the comparisons, the speech recognition scores (assessing the primary outcome of cochlear implantation) and quality of life were studied, as obtained before the intervention (with the patients’ own conventional hearing aids or BTEs) and 12 months post-implantation.

## Materials and methods

The pre-implantation and 12 months post-implantation results of adults with acquired severe/profound hearing loss, unilaterally implanted, were analyzed. All included patients in the database consecutively received a Nucleus CI at the Radboudumc, Nijmegen (The Netherlands), between 2010 and 2016. To be considered for cochlear implantation, the hearing loss had to be severe to profound and the obtained speech recognition measured with a well-adjusted (eventually refitted) conventional BTE hearing aid-had to be less than 70% phoneme score (which equals 44% word score).

The inclusion criteria for this retrospective analysis were age at implantation > 17 years and postlingual onset of hearing loss. The exclusion criteria were abnormal anatomy of the cochleovestibular system and known psychiatric diseases. This resulted in a study group of 164 adult CI recipients (71 males and 93 females). The audiological data consisted of the unaided audiometric thresholds of both ears and aided speech recognition scores, obtained with Dutch lists of monosyllables (NVA word lists; Nederlandse Vereniging van Audiologie), presented in the sound-fields at 65 dB SPL [24].

Quality of life data were measured with the standardized and validated Nijmegen Cochlear Implant Questionnaire, NCIQ [25, 26]. The NCIQ is a questionnaire consisting of six domains related to hearing loss: basic hearing perception, advanced perception, speech production and the psychosocial domains self-esteem, activity limitation en social interactions. The questionnaires were sent by post and returned after the questionnaire was filled in. The response rate was 58% pre-implantation and 68% post-implantation, which is an acceptable response rate for questionnaires [27].

In Table 1, patient characteristics are listed. Audiological data were measured using standard audiological equipment (using THD-39 headphones) and standard audiological procedures. The audiological equipment was calibrated according to the ISO 389 standard. Measurements were carried out in double-walled sound-attenuated booths complied with the ANSI 3.1. standard. For the sound-field measurements (speech recognition testing), the loudspeaker was positioned at 1.5 m distance in front of the patient.

**Table 1:** Patient characteristics

Patient characteristics			
Male	71		43%
Female	93		57%
Mean age at implantation	62		SD (14)
Audiometric characteristics			
Pre-implantation hearing aid use, *N* (%)	Ear to be implanted: 55 (34%)		Contralateral ear: 34 (21%)
Mean thresholds (dB HL) pre-implantation (SD)			
PTA3 (mean threshold 0.5, 1,2 kHz)	99 (14)		92 (18)
PTA4 (mean threshold 0.5, 1, 2, 4 kHz)	102 (13)		95 (17)
Etiology			
Unknown		72	
Congenital hearing loss (incl. rhesus antagonism1, Rubella2)		4	
Syndromal hearing loss (Crest8, Melas1, Meniere5, Usher7)		22	
Hereditary		52	
Acquired (meningitis1, otitis1, ototoxic medication2, meningioma2, trauma1, otosclerosis2, sudden deafness4, mumps virus1)		14	

### Data analyses

Based on the pre-implantation audiometric thresholds and speech recognition scores of the ear to be implanted, CI recipients were divided into two groups: those that fulfilled the conservative criteria at implantation (mean hearing loss at 0.5, 1 and 2 kHz (referred to as PTA3) > 85 dB HL and phoneme scores with hearing aids < 30%), the inside conservative criterion group (IC), and the outside conservative criterion group (OC). The latter group comprised all CI users that fulfilled the broadened criteria but not the conservative criteria.

### Research question

The two research questions that were studied were

1. Is the 1-year post-implantation speech recognition score of the expanded criteria group (OC group) comparable with the 1-year post-implantation speech recognition score of the conservative criteria (IC) group?
2. Is the 1-year post-implantation quality of life score of the OC group comparable with the 1-year post-implantation quality of life score of the IC group?

### Statistical analyses

The differences between the OC and IC subgroups were, if applicable, statistically (IBM^©^ SPSS^©^ Statistics for Windows, version 22) tested using the Student *T* test or the Welch (*t* test of unequal variances). Average scores are presented as mean (± standard deviation). In addition, if there was no normal distribution of the data, the bootstrap method was applied.

## Results

**Fig. 1:**
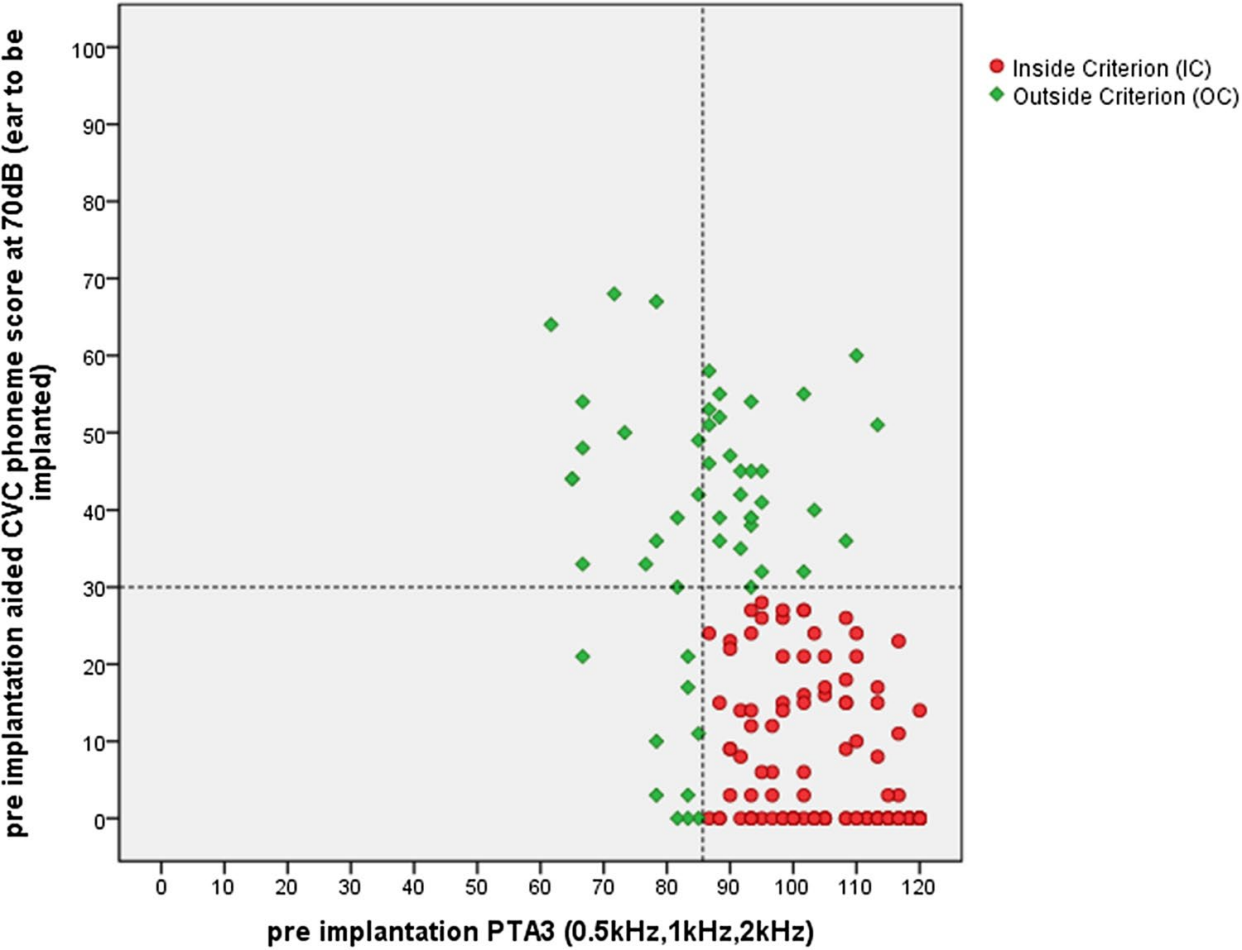
The pre-implantation hearing loss levels of 164 CI recipients, presented and classified according to the indication criterion. The horizontal axis shows the pre implantation PTA3 score of the ear to be implanted. The vertical axis shows the aided phoneme scores. Dashed lines indicate the indication boarders

In Fig. 1, the pre-implantation hearing loss levels of the 164 CI recipients are presented and classified. The horizontal axis shows the pre-implantation PTA3 score of the ear to be implanted. The vertical axis shows the aided phoneme scores of the ear to be implanted. Each dot represents one patient. Red dots refer to patients falling within the IC group and green dots indicate patients that fall within the OC group.

*Question 1*. Is the 1-year post-implantation speech recognition score of the IC group comparable with that of the OC group?

As the bootstrap method showed comparable results, the results of the *t* tests are reported below. The OC group showed a higher phoneme score 1-year post-implantation compared to the IC group (respectively, phoneme score of 84% and 78%, which equal word scores of, respectively, 67% and 56%). This difference was significant (*t* = − 3.29; *df* = 149; *p* = 0.001; 95% CI 2.57; 10.33), suggesting that the relaxed criteria have a positive influence on the speech recognition results post-implantation. Table 2 shows the mean speech recognition scores of the two groups.

**Table 2:** The average 1 year post-implantation phoneme score for the inside (IC) and outside (OC) criterion groups

Criterion	*N*	Mean %	SD
Phoneme scores			
Inside (IC)	112	78^a^	15
Outside (OC)	52	84^a^	10

^a^Significant (*p* = 0.001) difference between inside and outside criterion groups

*Question 2*. Is the 1-year post-implantation quality of life score of the IC group comparable with that of the OC group?

The average changes (and standard deviation) of the NCIQ domain scores are given in Table 3. Overall, in all domains of the NCIQ, a significant (*p* < 0.05) improvement after 1-year CI use was found.

**Table 3:** Changes in the domain scores of the NCIQ after Cl. Results of the IC group are compared to the results of the OC group

Benefit scores (pre–post)^a^			
Criterion	*N*	Mean	SD
Sound perception basic			
Inside	58	41	24
Outside	25	35	20
Speech production			
Inside	58	17	21
Outside	25	14	17
Sound perception advanced			
Inside	58	32^b^	19
Outside	25	22^b^	21
Self-esteem			
Inside	58	21	16
Outside	25	20	17
Activity limitation			
Inside	58	30	20
Outside	25	28	20
Social interaction			
Inside	58	30	19
Outside	25	26	19

^a^Higher scores indicate higher benefit

^b^Significant (*p* = 0.045) difference between inside and outside criterion

Comparing the difference score (pre–post) between the inside and outside group, there is a trend in which the inside criterion groups improve a little more than the outside group; however, this difference is only significant in the advanced perception domain (*t* = 2.07; *df* = 43; *p* = 0.045; 95% CI 0.25; 19.66). This is expected since patients that fall within IC group have a more severe hearing loss and a worse speech understanding pre-implantation and thus had more “room for improvement” with CI than patients with better hearing pre-implantation.

### Correlation between the improvement in speech understanding and the NCIQ perception

#### Scores

**Fig. 2:**
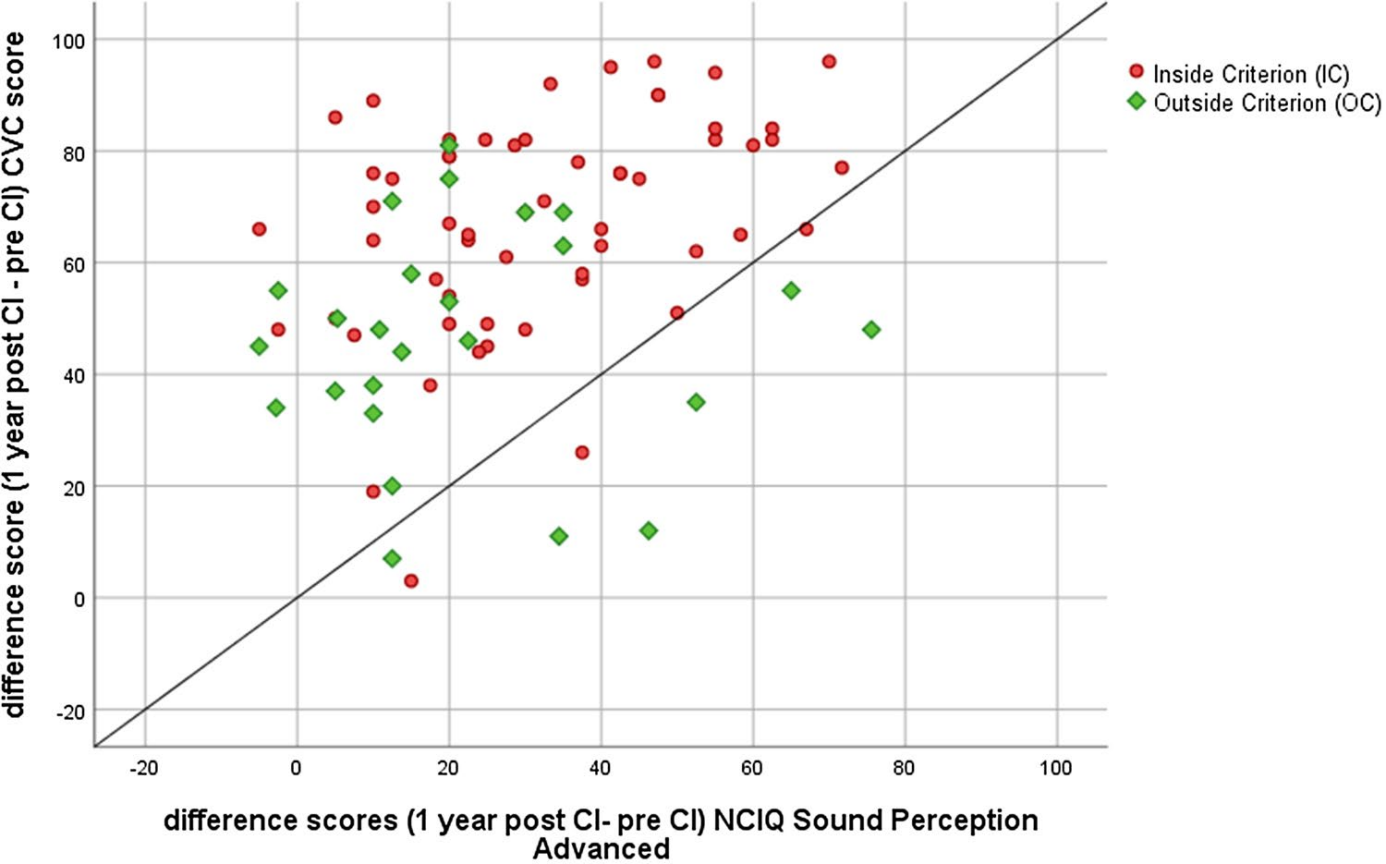
The relation between difference scores (the 12-month post-implantation minus the pre-implantation scores) regarding the phoneme score (*Y*-axis) and the NCIQ subdomain sound perception advanced (*X*-axis)

In Fig. 2, the relation between the difference scores (12 months post–pre-implantation) of the phoneme score and the NCIQ score of the subdomain sound perception advanced is presented. The correlation is statistically significant (*r* = 0.035, *p* = 0.001, two tailed), indicating a consistency between the measured data and the patient experiences.

## Discussion

The results described above endorse the idea that in countries that hold on to more conservative criteria for CI candidacy, expansion of CI indication is beneficial for long-term benefit [2–4, 28, 29]. CI performance has improved and more evidence was found for the positive effect of residual hearing on CI outcome, strengthening the growing need for a shift of CI indication. In this study, we observed that patients that fall within conservative inclusion criterion (IC) obtain a lower speech understanding score with a cochlear implant than CI recipients that fall within the expanded criteria group (OC). This result supports the idea that timely implantation may lead to better speech understanding with a CI (as reported by Snel-Bongers et al. [16]). Since hearing loss might deteriorate over time, excluding patients with severe hearing loss will imply a delay rather than an irrevocable refusal for CI. To this end, audiologists and otolaryngologists should be aware of the irreversible consequences of poor speech perception. Poor speech perception leads to poor communication, which has a devastating effect on an individual’s quality of life [8].

Concerning quality of life, it was expected that patients falling within the conservative inclusion criteria would experience a higher benefit from cochlear implantation than the patients with some functional residual hearing (outside criterion group). This was indeed the case concerning the advanced speech perception domain; however, not with regard to speech production and the patients’ psychosocial functioning. In these domains, the benefit was found to be comparable.

The speech recognition results are in line with the literature, suggesting that waiting too long before CI might increase the risk of auditory deprivation. It remains debatable what the exact criteria should be to justify both residual hearing on the one side and need for improved hearing on the other side. Based on the literature [3, 7, 8, 16] and on our clinical experience, the selection of candidates for cochlear implantation is a multi-factorial process and thus needs a multidisciplinary approach. The audiological criterion might be considered as a general guideline that should be applied more or less strict, depending on the accompanying medical, social, and personal characteristics of an individual.

As the present study focuses on the indication criteria for bilateral severe to profound hearing loss, the results do not reflect indication criteria for single-sided deafness (SSD) or asymmetric hearing loss (AHL). However, even though SSD and AHL are not the scope of this study, the known effect of non (or too late)-treatment of SSD or AHL [12] should be mentioned; specifically because in most countries, SSD and AHL remain untreated in the vast majority of patients. In general, the level of evidence for the effect of cochlear implantation in SSD and AHL is low; this is mainly due to the large variation between SSD/AHL studies. To this end, Van de Heyning et al. [30] developed, in consensus with expert panels, a protocol for the assessment of treatment options and outcomes in recipients with SSD and AHL, aiming at harmonizing assessment methods across centers and at generating a growing body of high-level evidence for those treatment options. The authors describe literature that provides evidence that cochlear implantation in SSD or AHL improves speech perception in noise, sound localization, quality of life and decreases the severity and incidence of tinnitus. Although cochlear implantation might be a treatment for (incapacitating) tinnitus, literature shows that cochlear implantation can have both a positive effect on tinnitus (decreased complaints) and a negative effect on tinnitus (a temporary or permanent induction of tinnitus) [31–33]. This and other recent studies improve knowledge on (long term) treatment of SSD/AHL and tinnitus which might be useful to guide future CI candidates [34, 35].

A limitation of this study is the retrospective study design, causing a risk of bias and confounding. A second limitation is the fact that we did not systematically test the effect of bimodal fitting, but only included the best aided condition post-implantation, based on our focus on the actual hearing situation of CI recipients.

Furthermore, it should be noted that in some countries, the CI indication has recently been reconsidered (e.g. the UK) or is not as strict as the criterion for non-aided PTA and aided speech recognition applied in the present study. Nevertheless, the present retrospective data substantiate the importance of timely intervention in adults with some residual hearing to improve their communication skills. This result might stimulate a critical evaluation in case of conservative CI indication.

Summarized, the data above suggest that the expansion of indications has a long-term positive effect on the speech recognition performance of patients with severe hearing loss. It affects quality of life positively (owing to an earlier change from BTE to CI). In several countries, such patients are currently not considered for cochlear implantation. The benefit relates to communication skills and the subjective day-to-day functioning in society.
